# Dragon’s blood secretion and its ecological significance

**DOI:** 10.1007/s00049-016-0212-2

**Published:** 2016-03-19

**Authors:** Joanna Jura-Morawiec, Mirela Tulik

**Affiliations:** Polish Academy of Sciences Botanical Garden - Centre for Biological Diversity Conservation in Powsin, Prawdziwka 2, 02-973 Warsaw, Poland; Department of Forest Botany, Warsaw University of Life Sciences-WULS, Nowoursynowska 159, 02-776 Warsaw, Poland

**Keywords:** Resin, Latex, Laticifers, Constitutive defence, Induced defence

## Abstract

Dragon’s blood is the name given to a red exudate produced by some plant species belonging to the genera *Daemonorops*, *Dracaena*, *Croton* and *Pterocarpus*. These are endemic to various parts of the globe. It is classified as a resin or latex depending on its mode of secretion and its chemical composition, which is species specific. This red substance functions in defence and is produced (a) constitutively and stored in preformed anatomical structures, or (b) by induction in response to traumatic events, such as mechanical injury, pathogen attack or invasion by insects. Apart from its defensive role in plants, dragon’s blood is also a valuable natural resource renowned since antiquity for its diverse medicinal properties and uses in art. Despite the great importance of dragon’s blood, our knowledge of the biological basis for its secretion is still incomplete. This review summarizes recent advances in the study of the anatomical basis for its secretion, and discusses its classification and ecological function. Bringing some clarity to these issues may also help in the commercial sourcing of dragon’s blood.

## Introduction

During the course of plant evolution, adaptation to biotic and abiotic stresses is often accompanied by modification of the organism on a range of different levels of organization that may involve changes to its morphology, physiology and biochemistry. Secretion of resin or latex is only one of many defence mechanisms that protect the plant against insect invasions or pathogen attacks (Langenheim 2003). Amongst the angiosperms occurs a small group of plants called dragon’s blood trees that have the ability to produce a red substance referred to as dragon’s blood, which has ecological properties. This group includes both monocotyledonous and eudicotyledonous species belonging to the genera *Daemonorops*, *Dracaena*, *Croton* and *Pterocarpus* (Gupta et al. 2008). Dragon’s blood trees are endemic to various parts of the globe. The monocots *Daemonorops* spp. and *Dracaena* spp. are native to Southeast Asia, and to Socotra, Canary Islands, Madeira and Morocco, China and some countries of Southeast Asia, respectively (Roskov et al. 2015; http://e-monocot.org/), while the eudicots *Croton* spp. and *Pterocarpus* sp. are native to the countries of Central and South America (Weaver 1997; Wiersema and Leon 2013; Roskov et al. 2015). A list of species of dragon trees has recently been included in a survey by Gupta et al. (2008). Dragon’s blood is variously classified as resin or latex, and may be produced by cells of the stem, leaves or fruit (Table 1), taking the form of drops or chips (Balfour 1883).

**Table 1:** Botanical sources of dragon’s blood and its contribution to plant defence mechanism

Family	Genus	Species	Origin	Plant defence
Monocots				
Arecaceae	*Daemonorops* spp.	6	Fruit	Constitutive
Asparagaceae	*Dracaena* spp.	3	Stem, leaf	Induced
Eudicots				
Euphorbiaceae	*Croton* spp.	8	Stem	Constitutive
Fabaceae	*Pterocarpus* sp.	1	Stem	Constitutive

The number of species after Gupta et al. (2008)

Besides its great importance for the plants that produce it, dragon’s blood is also a natural resource valuable for humans. Its antiviral, antibacterial and antifungal properties have been known since antiquity (reviewed by Gupta et al. 2008). Currently, the antioxidant properties of its extract are used by the cosmetic industry in the production of anti-ageing skin creams. Studies are also being conducted to verify its anti-cancer properties (e.g. Rossi et al. 2003; Lopes et al. 2004; Gonzalez and Valerio 2006). The increasing demand of this natural resource has resulted in the overexploitation of dragon’s blood-producing trees, and this is one of the factors that have adversely affected the size of their populations. Mainly, this affects *Dracaena* spp. By now, both *Dracaena draco* and *D. cinnabari*, the original sources of dragon’s blood have been included in the IUCN (International Union for Conservation of Nature) Red List of Threatened Species [http://www.iucnredlist.org/], and *D. cochinchinensis*, which is a main source of red resin in China, has been recognized as an endangered species in that country (Anon 1987 after Wang et al. 2011). Although dragon’s blood and its properties have been known since ancient times, our knowledge of the anatomical basis for its secretion by plants remains incomplete. One of the reasons for this may be the restricted distribution of dragon’s blood trees. The aim of this article is to summarize the current state of knowledge regarding the anatomical and ecological aspects of dragon’s blood secretion. Bringing some clarity to these issues may also help in the commercial sourcing of dragon’s blood.

## The anatomical basis for the secretion of dragon’s blood

**Fig. 1:**
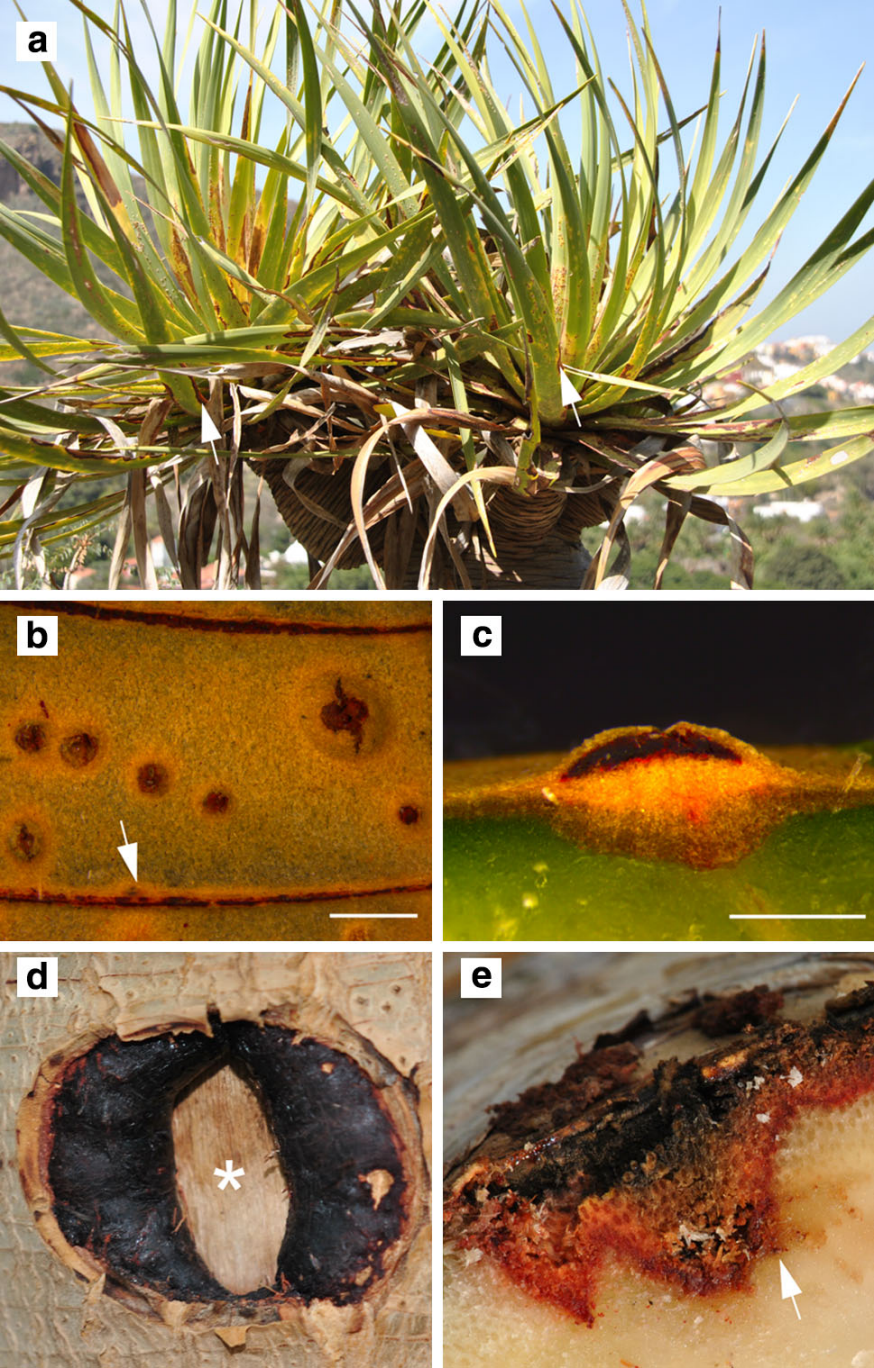
Dragon’s blood of *Dracaena draco*. **a** Dragon’s blood tree growing at Jardín Botánico Canario "Viera y Clavijo" with leaves infected by *Cochinilla algodonosa*, note the* reddish-brown* spots on their surface (*arrows*). **b** Leaf scar (*arrow*) and lenticels filled with resin on stem surface. **c** Cross section of a lenticel. **d** Stem wound with margins covered with resin, the central, dead part is marked with an *asterisk*. **e** Cross-sectional view of a stem wound; the dragon’s blood forms a barrier that isolates the infected tissue from healthy tissues. *Scale bar* **b**, **c** = 1 mm

Secretion and storage of resin in conifers, as well as latex in eudicotyledonous plants, both herbaceous and tree species, are usually associated with the formation of specialized anatomical structures, such as resin ducts and laticifers, respectively. In the stem of monocot arborescent plants of the genus *Dracaena*, however, these special secretory structures are absent (Fan et al. 2008). Instead, dragon’s blood is produced by cells of the parenchymatous ground tissue surrounding the primary and secondary vascular bundles, as well as by cortex cells located adjacent to the secondary protective tissue (Jura-Morawiec and Tulik 2015). These cells have no specific morphological/anatomical traits and can currently be identified only on the basis of their red-coloured contents. The secretion of dragon’s blood in stems of *D*. *cochinchinensi*s has been observed only in individual plants no younger than 30–50 years (Wang et al. 2010a). In *D.draco*, the onset of secretion is not determined by age, and thus, dragon’s blood is produced by young stems (Jura-Morawiec and Tulik 2015). Red exudate has also been detected in leaf cells of *D*. *cochinchinensis*, *D*. *cambodiana* (Wang et al. 2010b; Ou et al. 2013) and in *D. draco* (Fig. 1a); however, the structural basis for its secretion has not yet been described.

In the genus *Croton*, dragon’s blood is secreted by nonarticulated laticifers and/or nonspecialized parenchyma cells (Rudall 1987; Farias et al. 2009). The former are more numerous in the phloem, whereas the latter are abundant in the cortex. Laticifer abundance varies in *Croton* spp. and is determined by (a) the age of the plant, (b) its position on the tree, and (c) the environment. In general, laticifers are less abundant in old than in young stems (Rudall 1994); the branches have greater laticifer densities than the stem, and trees of the tropical rain forest have more laticifers than those of semi-deciduous tropical forests (Farias et al. 2009).

Nowadays, most dragon’s blood for commercial use is gathered from immature fruits of rattan palms of the genus *Daemonorops* (Pearson and Prendergast 2001; Baumer and Dietemann 2010). However, neither the anatomical basis for its secretion in this species, nor for the stem of *Pterocarpus* sp. has yet been described in the literature.

## Dragon’s blood: resin, latex or sap?

According to Langenheim (2003) resin “is a lipid-soluble mixture of volatile and non-volatile terpenoid and/or phenolic secondary compounds that are usually secreted in specialized structures located either internally or on the surface of the plant and are of potential significance in ecological interactions”. By contrast, latex, is a mixture of terpenoids, phenolic compounds, acids, carbohydrates, etc. having a protective role (Lewisohn 1991) and produced in special cells called laticifers (Fahn 1979). Chemical characterization of dragon’s blood is species specific and has been undertaken by many authors. For example, it is possible to distinguish between dragon’s blood from some individual species used in works of art, since it has been sold as a colourant for many centuries (Baumer and Dietemann 2010). Dragon’s blood of *Croton* spp. is usually referred to as latex due to the fact that it is secreted and stored by laticifers, and its major constituents are polymeric anthocyanidins, which co-occur with many minor constituents, including diterpenes and simple phenols (Salatino et al. 2007). Dragon’s blood secreted by stems of *Pterocarpus officinalis* is also called latex (Weaver 1997; Guerrero and Guzman 2004); however, information about the chemical composition of the exudate and its ecological function is poorly known. Dragon’s blood derived from species of *Dracaena* and *Daemonorops* is a phenolic resin (Langenheim 2003), with well-recognized chemical content (e.g. Gonzalez et al. 2000; Shen et al. 2007; Sousa et al. 2008). Sometimes, dragon’s blood is referred to as sap (e.g. Philipson 2001). However, this could prove to be a source of confusion, since plants produce other exudates referred to by that name, such as xylem sap and phloem sap, which are entirely different in terms of their location, chemical composition and function. Xylem sap is transported along xylem vessels and tracheids, is mainly composed of water, and contains several other components such as hormones and minerals, whereas phloem sap flows along sieve tubes (in angiosperms) and contains sugars, amino acids, hormones and minerals dissolved in water (Zimmermann and Brown 1971).

## Red latex/resin as a plant defence strategy

Dragon’s blood may act in constitutive or induced plant defence directed against pathogens, as well as against pests (Table 1). As mentioned above, the chemical composition of dragon’s blood is species specific, and is thus likely to vary greatly with respect to the type of insects/pathogens that may attack a given species of dragon’s blood tree. In *Croton draco*, dragon’s blood is found in laticifers of the cortex and phloem, and represents a constitutive (preformed) defence. Since the bark of *C. draco* is very thin, it provides little mechanical resistance, and dragon’s blood, together with oils and tannins probably act as effective deterrents against sucking insects and pathogens (Farias et al. 2009). In *Pterocarpus* sp., dragon’s blood flows freely when the bark is cut (Allen 1964), and therefore, one might expect it to play a role in constitutive plant defence. Dragon’s blood which coats the immature fruits of *Daemonorops* spp. also seems to be a part of a constitutive defence strategy. Lev-Yadun et al. (2009) pointed out that the red colour of unripe fruit can serve as a warning system, deterring herbivores from consuming chemically or physically defended fruit, and thus, their still immature seed. Therefore, it is likely that dragon’s blood is aposematic (sensu Lev-Yadun and Gould 2009; Lev-Yadun 2014). Moreover, by being sticky, it may physically and passively entrap small organisms and, owing to its toxic nature, kill them (Konno 2011). Possibly, it can also protect against water loss and high temperatures (Langenheim 2003).

In *Dracaena* spp., dragon’s blood secretion can be considered an induced natural defence mechanism, i.e. trauma is essential for triggering its formation. This can be due to mechanical injury, insect attack or pathogen infection (Fig. 1). Also localized tissue damage resulting from natural developmental processes, such as leaf drop or the formation of lenticels, may also induce dragon’s blood secretion, as in *D. draco* (Fig. 1b, c). Moreover, it has been shown that infection with the pathogenic fungi *Fusarium* (Wang et al. 2010a, 2011; Jiang et al. 2003), *Gibberella* and *Septoria* (Cui et al. 2013) stimulates the production and accumulation of resin in *Dracaena* spp. Following a traumatic event, dragon’s blood is synthesized and accumulates in the cells that border the wound or infected tissues. In this way, it helps prevent the spread of the pathogen and acts as a barrier between the wound/infected cells and healthy tissues (Fig. 1e) (sensu Shigo 1984). During the process of wound repair, dragon’s blood coats the margins of the wound (Fig. 1d) providing additional protection, possibly against desiccation. The red colouration indicating the presence of resin appears about two weeks following the wounding of a *D. draco* stem (Jura-Morawiec and Tulik 2015). This indicates that at first, the plant relies mainly on constitutive bark defences in the form of polyphenolic inclusions or calcium oxalate crystals (Nagy et al. 2000; Nakata 2012), both of which have been documented for *Dracaena* spp. (Prychid and Rudall 1999; Jura-Morawiec and Tulik 2015).

## Summary

Dragon’s blood secretion is a specialization occurring in a small group of plant taxa. Its composition and mode of secretion combine to produce effective defence mechanisms that have evolved along different pathways in species distributed across the globe. For some species, this feature has become a double-edged sword. On the one hand, it provides natural defence, but on the other, it makes the plant vulnerable to exploitation. Considering that some dragon’s blood tree species are in decline, it becomes increasingly important that the secretion of dragon’s blood is understood to help establish a sustainable harvest of red resin/latex for commercial use.
